# *In Vitro* Evidence for Immune-Modulatory Properties of Non-Digestible Oligosaccharides: Direct Effect on Human Monocyte Derived Dendritic Cells

**DOI:** 10.1371/journal.pone.0132304

**Published:** 2015-07-06

**Authors:** Sarah Lehmann, Julia Hiller, Jeroen van Bergenhenegouwen, Leon M. J. Knippels, Johan Garssen, Claudia Traidl-Hoffmann

**Affiliations:** 1 Institute of Environmental Medicine, UNIKA-T, Technische Universität München, Augsburg, Germany; 2 Center of Allergy and Environment (ZAUM), Member of the German Center for Lung Research (DZL), Technische Universität & Helmholtz Zentrum München, Munich, Germany; 3 Nutricia Research, Department of Immunology, Utrecht, The Netherlands; 4 Division of Pharmacology, Utrecht Institute for Pharmaceutical Sciences, Faculty of Science, Utrecht University, Utrecht, The Netherlands; 5 CK CARE-Christine Kühne Center for Allergy Research and Education, Davos, Switzerland; University of Bergen, NORWAY

## Abstract

Infant formulas containing non-digestible oligosaccharides (NDO) similar to the composition in breast milk or a combination of lactic acid bacteria (LAB) and NDO have been shown to harbor preventive effects towards immune-regulatory disorders. The aim of this study was to investigate the immune-modulatory potential of non-digestible short chain galacto- and long chain fructo-oligosaccharides (scGOS/lcFOS) mimicking the natural distribution of oligosaccharides in human breast milk in presence or absence of certain LAB strains in human monocyte derived dendritic cells (MoDC). Immature human MoDC prepared from peripheral blood of healthy non-atopic volunteers were screened *in vitro* after stimulation with specific scGOS/lcFOS in presence or absence of LAB. IL-10 and IL-12p70 release was analyzed after 24 hours in cell-free supernatants by enzyme-linked immunosorbent assay (ELISA). A luminex-based assay was conducted to assess further cytokine and chemokine release by MoDC. To investigate the resulting T cell response, stimulated MoDC were co-incubated with naïve T cells in allogeneic stimulation assays and intracellular Foxp3 expression, as well as immune-suppressive capacity was determined. Oligosaccharides did not induce relevant amounts of IL-12p70 production, but did promote IL-10 release by MoDC. Furthermore, scGOS/lcFOS mixtures exerted a significant enhancing effect on LAB induced IL-10 secretion by MoDC while no increase in IL-12p70 production was observed. Blocking toll like receptor (TLR)4 abrogated the increase in IL-10 in both the direct stimulation and the LAB stimulation of MoDC, suggesting that scGOS/lcFOS act via TLR4. Finally, scGOS/lcFOS-treated MoDC were shown to upregulate the number of functional suppressive Foxp3 positive T cells following allogeneic stimulation. Our results indicate anti-inflammatory and direct, microbiota independent, immune-modulatory properties of scGOS/lcFOS mixtures on human MoDC suggesting a possible induction of regulatory T cells (Tregs). The tested combinations of LAB and scGOS/lcFOS might represent a useful dietary ingredient for the maintenance of intestinal homeostasis via the induction of Tregs.

## Introduction

Over the past decades predominantly western countries are facing an increasing occurrence of chronic inflammatory diseases associated with loss of tolerance such as allergies or inflammatory bowel disease. Accumulated evidence suggests a link between these immune-regulatory disorders and an imbalance in the intestinal microbiota also referred to as dysbiosis [1–3]. An adequate constitution of the intestinal microbiota and gut colonization in early stages of life is crucial for maintaining health as adult [4]. It has been shown that the infant’s immune system matures by exposure to the intestinal microbiota initiated by microbial colonization of the gut through exposure to bacteria during birth and early life [5,6]. Evidence exists that breastfeeding decreases the incidence of infections [7] and can be protective regarding allergic diseases [8,9]. Human milk plays an important role in the establishment of the microbiota and the maturation of the child’s immune system [10] through its unique composition involving antimicrobial properties, transfer of immunological and tolerogenic stimuli and its high content of non-digestible human milk oligosaccharides (HMO) [11,12]. Infant formula supplemented with a specific manufactured 9:1 mixture of short chain galacto-oligosaccharides and long chain fructo-oligosaccharides (scGOS/lcFOS), resembling the composition of HMO is associated with a reduced cumulative incidence of atopic dermatitis during the first six month of life. In a follow-up study, a protective effect on the incidence of some allergic manifestations could be observed after five years [13,14]. Moreover, a decreased occurrence of allergic manifestations at the age of two and a reduced number of infectious episodes beyond the intervention period have been reported in infants that received formula containing scGOS/lcFOS [15]. Additionally, other studies showed a beneficial effect on mucosal immunity showing higher concentrations of fecal secretory immunoglobulin (Ig)A [16,17], reduced levels of immunoglobulin free light chain, and a beneficial immunoglobulin profile in infants at high risk for allergy [18,19]. Overall, these data suggest that infant formula supplemented with this specific mixture of scGOS/lcFOS has immune-regulatory properties but much remains unknown about their mechanism of action. Besides the known prebiotic effects of NDO on modulating the composition of the intestinal microbiota [20,21] possible direct systemic effects of NDO on epithelial and immune cells are currently under investigation [22]. Naarding et al. showed that HMO have the capacity to bind to the C-type lectin receptor dendritic cell-specific intercellular adhesion molecule-3-grabbing non-integrin (DC-SIGN) on dendritic cells (DC) [23] and Capitan-Canadas et al. recently described a non-prebiotic effect of oligosaccharides on monocytes via activation of the pattern recognition receptor (PRR) Toll like receptor 4 (TLR4) in rats [24]. In a follow-up study, a TLR4-mediated direct effect of NDO on human intestinal epithelial cell lines could be demonstrated [25].

In this study, we analyzed the direct immune-modulatory effects of scGOS/lcFOS on MoDC. We show that MoDC stimulation with scGOS/lcFOS increases IL-10 release in the absence of IL-12 secretion. Furthermore, scGOS/lcFOS-treated MoDC were found to increase the number of Foxp3^+^ T cells. Overall scGOS/lcFOS might contribute to intestinal homeostasis via the induction of the anti-inflammatory cytokine IL-10 and the induction of Tregs.

## Methods

### Subjects and Ethic Statement

For this study peripheral blood of healthy, non-atopic donors was used. Volunteers were screened for total serum IgE levels and for specific IgE against common allergens as described before [26] and non-atopic individuals were defined by total IgE lower 100 kU. The ethical committee of Technische Universität München approved the study and all volunteers enrolled in the study had given their written informed consent.

### Bacteria Preparation

The *Bifidobacterium breve* strain NutRes200 as well as the *Lactobacillus rhamnosus* strain NutRes1 were used. *L*.*rhamnosus* was available as 0.5 ml cultures of 5.9x10^9^ cell counts stored at -80°C. Lyophilized *B*.*breve* was diluted in D-PBS (Gibco/Invitrogen) and a 25x10^9^ cfu/ml stock solution was prepared and stored at -80°C. To prepare bacterial strains for stimulation experiments, stocks were thawed, diluted appropriately in D-PBS (Gibco/Invitrogen) and concentrations of 1x10^5^-1x10^7^cfu/ml were generated by serial dilution in cell culture medium.

### Generation of Monocyte Derived Dendritic Cells

For the generation of monocyte derived dendritic cells (MoDC) PBMC were isolated from peripheral blood of healthy volunteers by density gradient centrifugation (Lymphoprep, Axis-Shield). CD14^+^ monocytes were purified by MACS (CD14^+^ beads, Miltenyi Biotec) and cultured in RPMI 1640 supplemented with 10% FBS (HyClone), 2 mmol/l L-glutamine, 20 mg/ml gentamycin (all from Invitrogen Life Technologies) and 50 μmol/l 2-mercaptoethanol (Fluka) at 37°C, 5% CO_2_ in the presence of 50 U/ml rhGM-CSF and 50 U/ml rhIL-4 (both Promocell). Immature MoDC harvested on day 5 were >95% pure as analyzed by FACS analysis (CD14^-^ CD1a^+^ HLA-DR^+^ CD80^low^ CD83^-^ CD86^low^ CD40^low^). MoDC were routinely checked for viability (propidium iodide staining).

### Stimulation of MoDC with scGOS/lcFOS and LAB

Immature MoDC were seeded into 96-well flat bottom plates (Nunc) at a density of 1x10^5^ cells/ml in complete DC medium and treated with the bacterial strains at concentrations of 1x10^5^-1x10^7^cfu/ml. Bacterial stimulation was performed either in the absence or presence of 5 mg/ml scGOS/lcFOS mixture (9:1; scGOS; Friesland Campina, lcFOS; Orafti). To block TLR4 activity, a TLR4 antagonist (5 μg/ml, LPS-RS Ultrapure, InvivoGen) was used. LPS (100 ng/ml, Sigma) served as positive control for MoDC stimulation indicating functional MoDC stimulation. Untreated MoDC were used as negative control. After 24 hours incubation at 37°C and 5% CO_2_, cytokines IL-10 (BD Biosciences) and IL-12p70 (eBioscience) were analyzed in cell-culture supernatants by ELISA according to the manufacturer’s protocol. Furthermore, a broader selection of MoDC released mediators were analyzed by a luminex-based assay conducted by the Multiplex Core Facility Laboratory for Translational Immunology, Department of Pediatric Immunology, University Medical Centre Utrecht. Expression of maturation markers CD80 (FITC-conjugated anti-human, BD), CD83 (PE-conjugated anti-human, eBioscience), CD86 (APC-conjugated anti-human, BD), CD40 (FITC-conjugated anti-human, eBioscience), HLA-DR (APC-conjugated anti-human, eBioscience) and viability were measured by flow cytometry after stimulation of MoDC.

### Allogeneic Stimulation Assay of Naïve T cells

The capacity of oligosaccharides-matured MoDC to instruct the polarization of T lymphocytes was examined performing allogeneic stimulation assays (ASA). After 24 hours scGOS/lcFOS-stimulated MoDC were washed and co-incubated with naïve CD4^+^CD45RA^+^ T cells at a DC:T cell ratio of 1:10 in T cell medium containing RPMI supplemented with 1% L-glutamine, 1.12% nonessential amino acids, 1.12% sodium pyruvate, 1% pen-strep (all from Gibco), 1% 5 mM mercaptoethanol and human serum (5%, Lonza) for 7 days. Allogeneic CD4^+^CD45RA^+^ T cells were isolated from PBMC of non-atopic donors using the CD4^+^CD45RA^+^ naïve T Cell Isolation Kit II (Miltenyi Biotech). A positive control for Treg polarization was generated by priming naïve T cells with 5 ng/ml rhTGF-β (Promokine), 200 U/ml rhIL-2 (Novartis), 5 μg/ml anti-IL-12 and 1 μg/ml aIFN-γ (both eBioscience) and co-culturing them with MoDC that have been incubated with medium only. 20 U/ml rhIL-2 was added to the cells on the third day of the ASA. After 7 days of co-culture cells were restimulated for 5 hours with phorbol 12-myristate 13-acetate/ionomycin (both Sigma Aldrich) in the presence of monensin (eBioscience) and brefeldin A (BD Biosciences). Dead cells were excluded using the LIVE/DEAD Fixable Aqua dead cell stain kit (Life Technologies) according to the manufacturer’s instructions, followed by surface staining of CD4 (APC-Cy7-conjugated anti-human, BD Biosciences). Cells were then fixed, permeabilized and stained for intracellular Foxp3 (APC-conjugated anti-human, eBioscience) using a Foxp3 staining kit (eBioscience) and analyzed by flow cytometry (FACSFortessa System, BD FACSDiva 7.0). The gating strategy of alive, CD4^+^, Foxp3^+^ T cells is shown in S1 Fig.

### Suppression Assay

To determine the functionality of potential regulatory effector T cells induced in the ASA, these cells were harvested on day seven of the ASA and co-incubated with allogeneic freshly isolated responder T cells (CD4^+^ T cell isolation Kit II, Miltenyi) at a ratio of 1:1 for five days in 96-well plates (BD) coated with anti-CD3 (1 μg/ml, BD). Soluble anti-CD28 (1 μg/ml, BD) and rhIL-2 (100 U/ml) were added. Responder T cells were stained with Cell Trace Violet Cell Proliferation Kit (Life Technologies) according to the manufacturers’ instructions at a final concentration of 6 μM and violet-positive cells were acquired on a FACS Fortessa (BD). The mix of ASA cells and violet stained responder CD4+ T cells was then stained (after five days of co-incubation) with CD4 APC-Cy7. Suppressive capacity was determined by setting gates on proliferated and non-proliferated cells and analyzing the ratio of these cell populations. The gating strategy of dividing/non-dividing CD4^+^ T cells is shown in S6 Fig.

### Statistical Analysis

Data are presented as mean ± SEM and statistically analyzed using the Mann-Whitney-Test or unpaired student’s t test, a *p* ≤ 0.05 was considered as statistically significant. Statistical analysis was conducted using GraphPad Prism software (version 6.0).

## Results

### scGOS/lcFOS Induce IL-10 Secretion of MoDC in the Absence of IL-12p70

**Fig 1:**
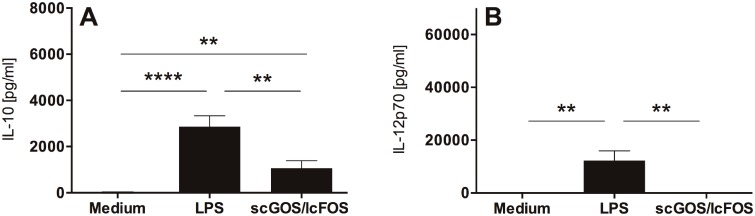
MoDC primed with scGOS/lcFOS are characterized by release of IL-10 while no IL-12p70 secretion is detectable. Immature human MoDC were incubated with scGOS/lcFOS (5 mg/ml) for 24h. Medium-treated MoDC served as negative control, MoDC matured by LPS as positive control for functional MoDC stimulation. Supernatants were analyzed for secretion of IL-10 (A) and IL-12p70 (B) by ELISA. Results are presented as mean ± SEM, ten independent experiments are shown. **** p<0.0001, ** p<0.01, unpaired student’s t test. scGOS/lcFOS = short chain galacto-, long chain fructo-oligosaccharides, MoDC = monocyte-derived dendritic cells.

To investigate whether scGOS/lcFOS can induce cytokine release by MoDC, immature human MoDC were stimulated with scGOS/lcFOS (5 mg/ml). The incubation of MoDC with oligosaccharides induced the release of IL-10 (Fig 1A), while no IL-12p70 release could be detected (Fig 1B). LPS-stimulated MoDC released both IL-10 and IL-12p70 (Fig 1A and 1B).

MoDC were phenotypically analyzed after incubation with either medium, LPS or oligosaccharides. Compared to medium, LPS induces maturation as measured by increased mean fluorescence intensities (MFI) specific for maturation markers CD80, CD83 and CD86. In contrast treatment with oligosaccharides only slightly upregulated maturation markers (S2 Fig). The viability of MoDC was not affected by LPS- or scGOS/lcFOS-stimulation (S2 Fig).

### scGOS/lcFOS Dose-dependently Activate TLR4

**Fig 2:**
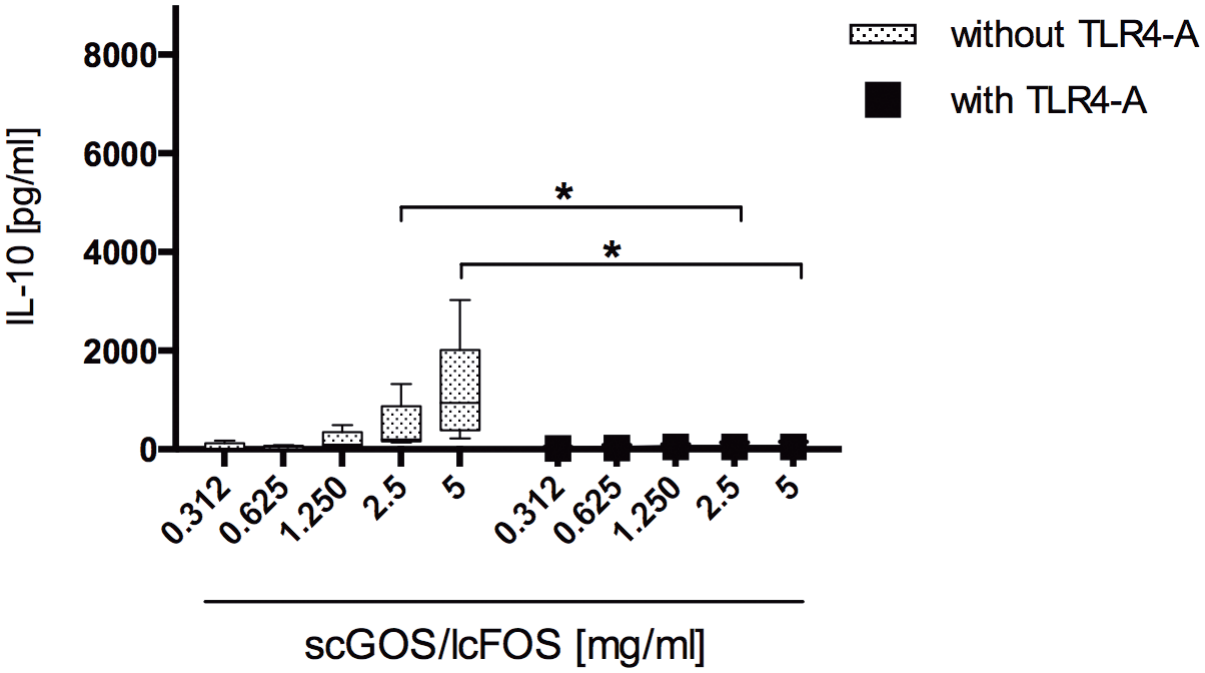
Stimulation with scGOS/lcFOS directly induces a dose dependent IL-10 release by MoDC that is impaired by addition of a TLR4 antagonist. Immature human MoDC were exposed to oligosaccharide mixture scGOS/lcFOS (312 μg/ml–5 mg/ml) for 24h in the presence (black marks) or absence (grey marks) of a TLR4 antagonist. The amount of IL-10 was measured by ELISA in cell-free supernatant. Results are presented as mean ± SEM, five independent experiments are shown. * p<0.05, Mann Whitney test. scGOS/lcFOS = short chain galacto-, long chain fructo-oligosaccharide-mixture, MoDC = monocyte-derived dendritic cells.

NDO were previously shown to stimulate TLR4 [25]. To investigate the potential involvement of TLR4 in the MoDC cytokine release following scGOS/lcFOS stimulation, we conducted a dose-response experiment in the presence and absence of a TLR4 antagonist. Stimulating MoDC with scGOS/lcFOS in the presence of the TLR4 antagonist completely abrogated MoDC IL-10 release (Fig 2, controls see S3 Fig).

**Fig 3:**
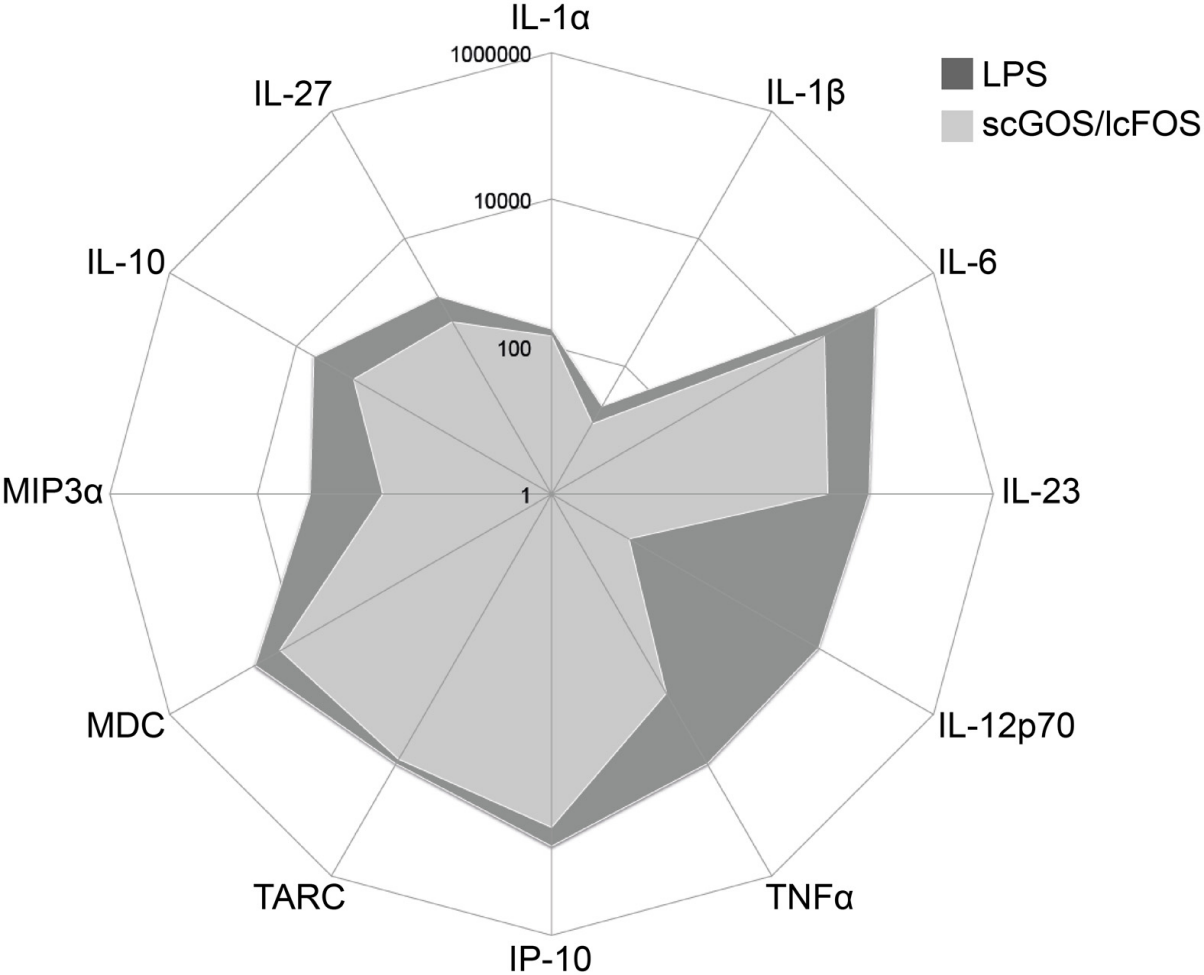
scGOS/lcFOS-stimulated MoDC release cytokines and chemokines in a different manner compared to LPS. MoDC were stimulated with scGOS/lcFOS (5 mg/ml) for 24h. MoDC matured by LPS served as positive control for functional stimulation. The amount of cytokines and chemokines in cell-free supernatants was measured by luminex-based assay (readout: IL-1α, IL-1β, IL-6, IL-23p19, IL-27, TNFα, IP-10, MDC (CCL22), TARC (CCL17), MIP3α (CCL20) and ELISA (IL-10, IL-12p70)). Results are presented in pg/ml. Three independent experiments are shown.

The observation that, in contrast to LPS, scGOS/lcFOS stimulated MoDC released IL-10 in the absence of IL-12 led us to consider whether other soluble mediators were also differentially affected. Upon comparing the LPS- with the scGOS/lcFOS-induced MoDC profile of mediator release several differences could be observed depending on the secreted mediator (Fig 3).

### scGOS/lcFOS Enhance LAB-induced IL-10 Release

**Fig 4:**
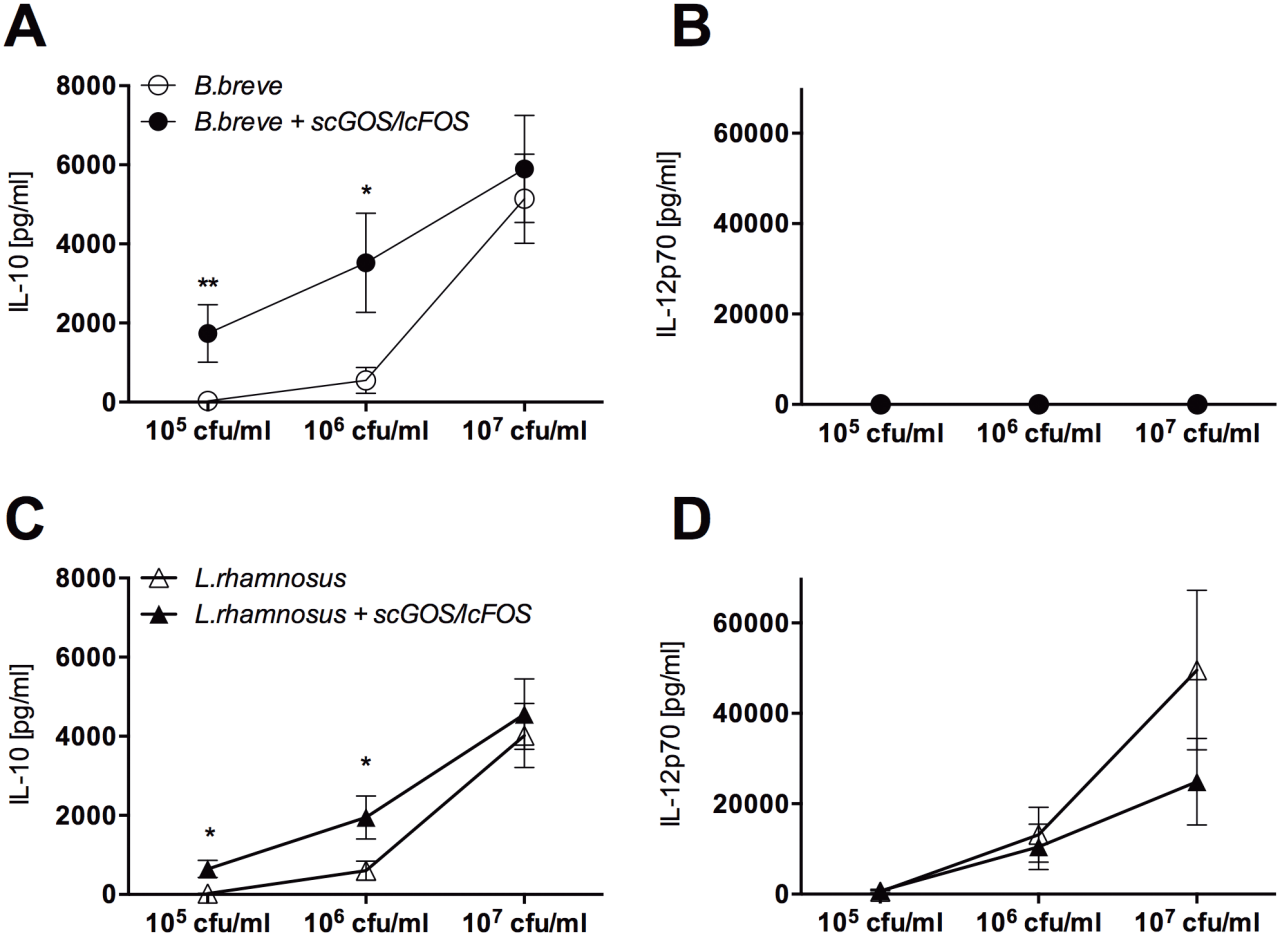
LAB-primed MoDC stimulated with scGOS/lcFOS are characterized by an enhanced release of IL-10 while no enhanced IL-12p70 secretion is detectable. Immature human MoDC were incubated with *B*.*breve* (clear dots, A, B) or *L*.*rhamnosus* (clear triangle, C, D) in different concentrations with (black marks) or without (clear marks) scGOS/lcFOS (5 mg/ml) for 24h. Supernatants were analyzed for secretion of IL-10 (A, C) and IL-12p70 (B, D) by ELISA. Results are presented as mean ± SEM, ten independent experiments are shown. * p<0.05, ** p<0.01, Mann Whitney test. LAB = Lactic acid bacteria, MoDC = monocyte-derived dendritic cells.

To examine how the presence of scGOS/lcFOS would affect LAB-induced MoDC cytokine release, MoDC were stimulated with either *B*.*breve* or *L*.*rhamnosus* in the absence or presence of scGOS/lcFOS. In contrast to *L*.*rhamnosus*, *B*.*breve* induced IL-10 release in the absence of IL-12p70 (S4 Fig). Presence of scGOS/lcFOS significantly enhanced IL-10 release for both strains while IL-12p70 release was not affected (Fig 4).

**Fig 5:**
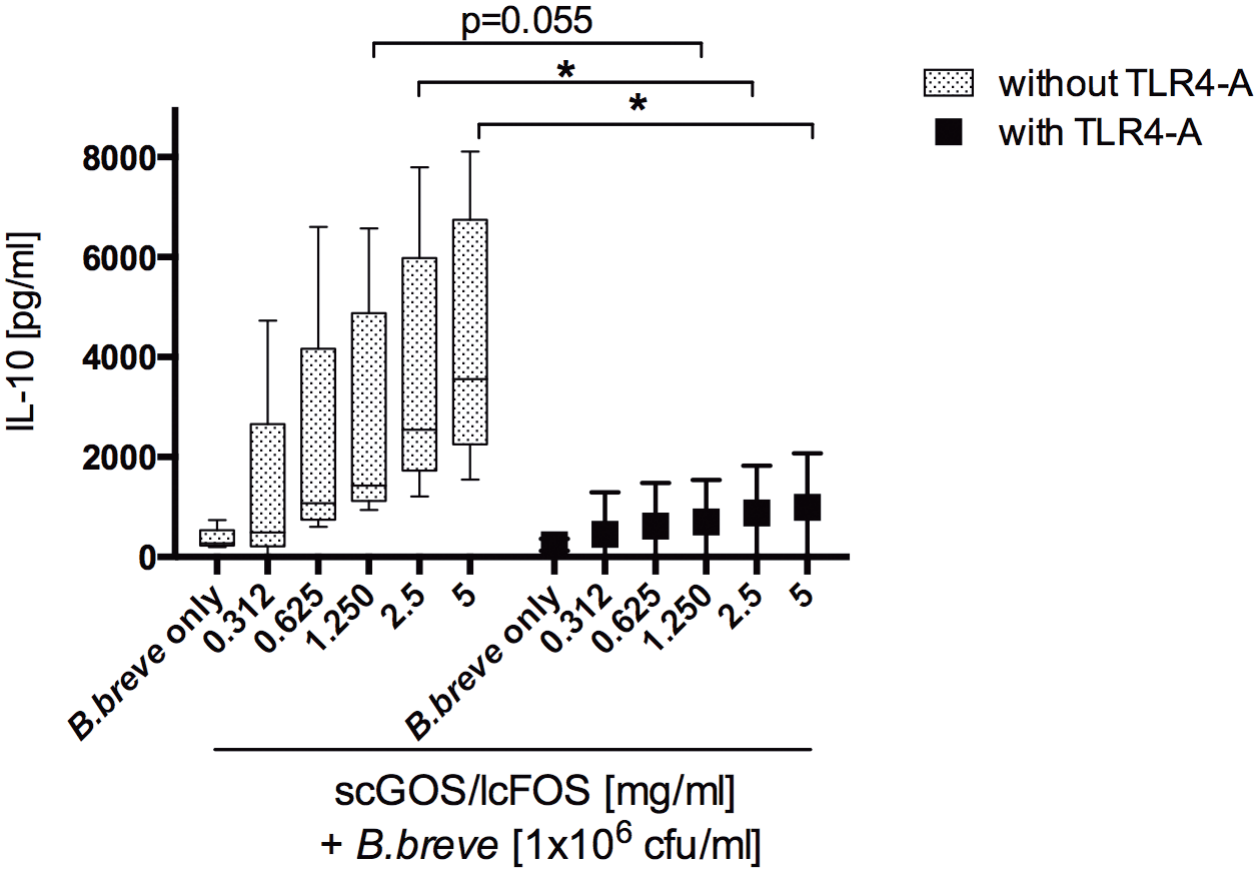
Stimulation with scGOS/lcFOS directly induces a dose dependent enhanced IL-10 release by LAB-primed MoDC that is impaired by addition of a TLR4 antagonist. Immature human MoDC were exposed to oligosaccharide mixture scGOS/lcFOS (312 μg/ml–5 mg/ml) in the presence of *B*.*breve* at a concentration of 1x10^6^cfu/ml in the presence (black marks) or absence (grey marks) of a TLR4 antagonist. The amount of IL-10 was measured by ELISA in cell-free supernatant after 24h. Results are presented as mean ± SEM, five independent experiments are shown. * p<0.05, Mann Whitney test. scGOS/lcFOS = short chain galacto-, long chain fructo-oligosaccharide-mixture, MoDC = monocyte-derived dendritic cells, TLR = Toll like receptor.

To investigate this differential effect on cytokine release in more detail, a larger selection of MoDC-derived soluble mediators was analyzed. scGOS/lcFOS was found to enhance mediator release depending both on the analyte measured and the bacterial strain used suggesting strain-dependent effects (S5 Fig). To investigate the potential contribution of TLR4 activity to the observed effect, MoDC were stimulated in the presence of a LPS antagonist. scGOS/lcFOS dose-dependently increased *B*.*breve-*induced MoDC IL-10 release. Blocking of TLR4-activity abrogated this enhanced effect (Fig 5).

### scGOS/lcFOS-Stimulated MoDC Induce Treg Development

**Fig 6:**
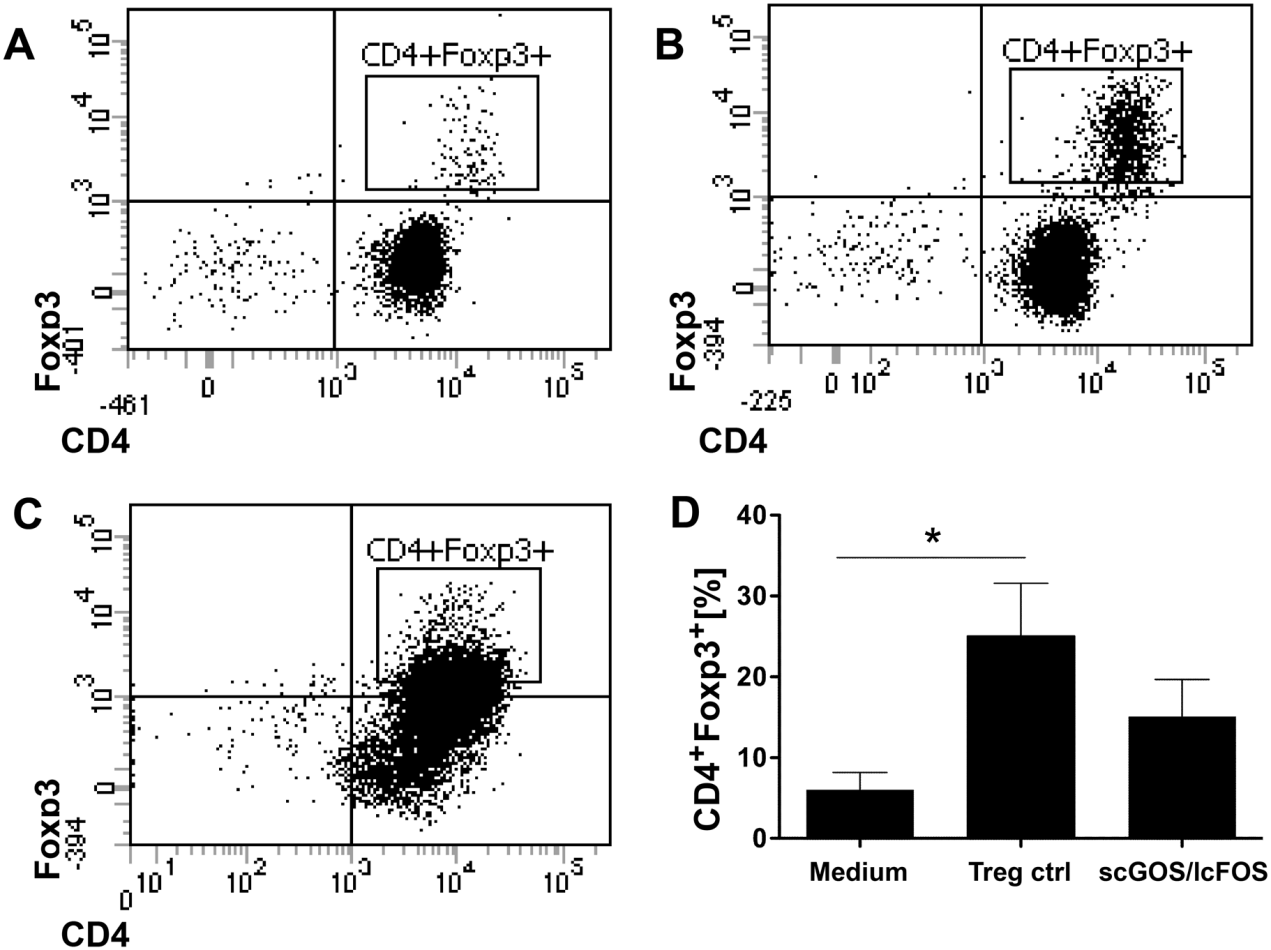
scGOS/lcFOS stimulated MoDC induce CD4^+^Foxp3^+^ T cells. MoDC stimulated with scGOS/lcFOS (5 mg/ml) were used to prime naïve CD4^+^CD45RA^+^ T cells in an ASA. At day 7, cells were stained for CD4 and intracellular Foxp3 was analyzed by flow cytometry. The dot plots show data from one representative experiment (A-C). T cells that have been co-incubated with medium-treated MoDC served as medium/negative control (A) and a positive control for Treg polarization is shown in (B). CD4^+^Foxp3^+^ T cells induced by scGOS/lcFOS stimulated MoDC are shown in (C). Data in (D) are expressed as percentages of CD4^+^Foxp3^+^ cells. Results are presented as mean ± SEM, 4 independent experiments are shown (D). * p<0.05, Mann Whitney test. ASA = allogeneic stimulation assay, scGOS/lcFOS = short chain galacto-, long chain fructo-oligosaccharides, MoDC = monocyte-derived dendritic cells.

DC release of IL-10 is associated with the induction of Tregs [27]. We therefore investigated whether the IL-10 release following stimulation of MoDC with scGOS/lcFOS would lead to induction of Tregs. MoDC were treated with scGOS/lcFOS for 24 hours, washed and subsequently co-cultured with naïve T cells in an allogeneic stimulation assay. Compared to control MoDC (medium-treated MoDC control Fig 6A, positive Treg control Fig 6B), scGOS/lcFOS-stimulated DC induced an upward shift in Foxp3 expressing T cells (Fig 6C). However, this increase did not reach significance (Fig 6D).

**Fig 7:**
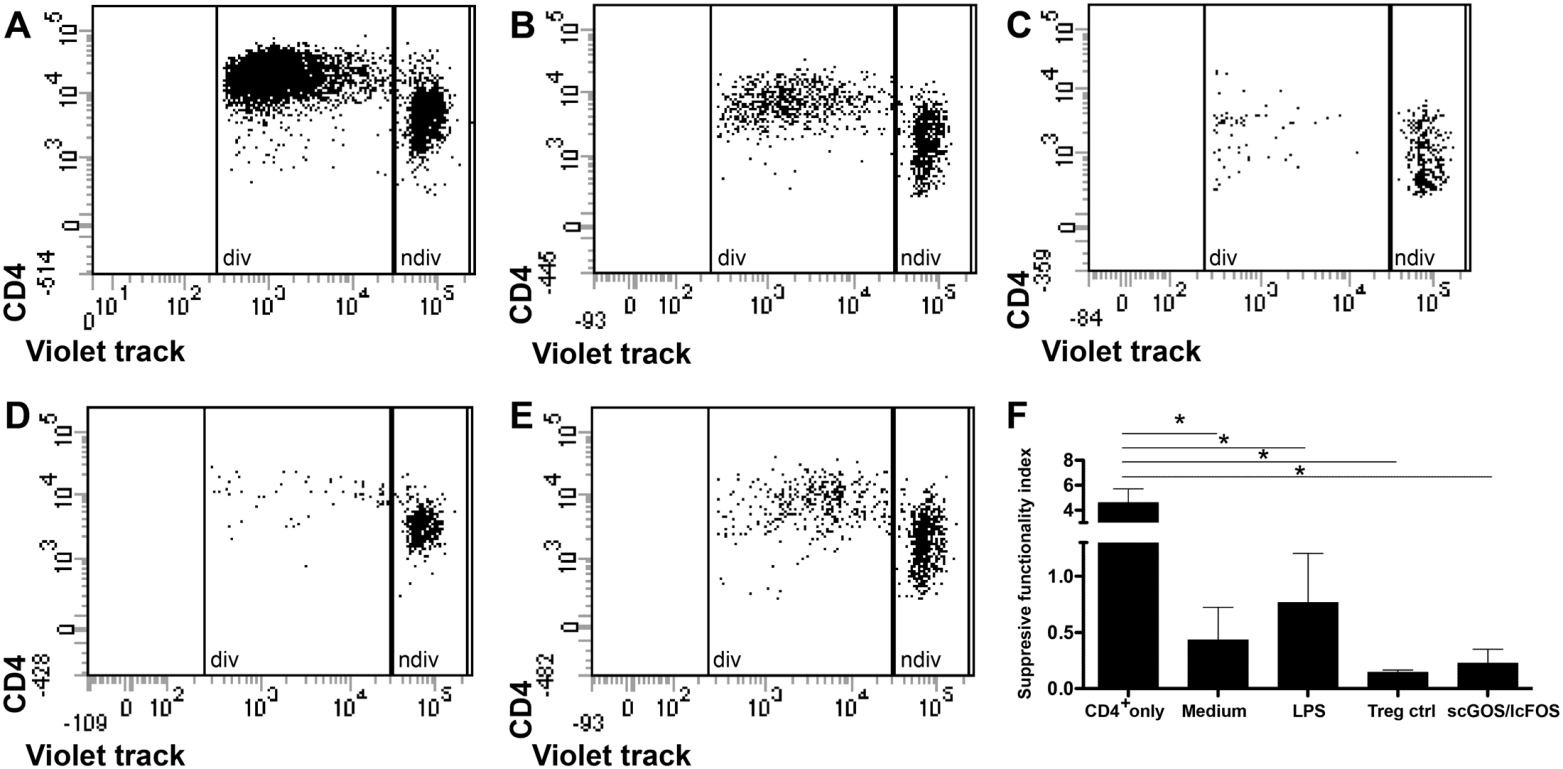
Suppressive capacity of T cells induced by scGOS/lcFOS-treated MoDC. T cells of allogeneic stimulation assays (ASA) were harvested on day seven and co-incubated with freshly isolated, violet-labeled responder CD4^+^ T cells a ratio of 1:1 for five days in presence of anti-CD3/28 and rhIL-2. The mix of ASA cells and violet stained responder CD4^+^ T cells was then stained (after five days of co-incubation) with CD4 APC-Cy7. The dot plots show data from one representative experiment. Proliferation of responder T cells only (A) or in co-incubation with T cells induced by medium- (B), LPS- (C) and scGOS/lcFOS- (E) stimulated MoDC and Treg control (D) is shown. Results in (F) are presented as mean ± SEM, 4 independent experiments are depicted. Proliferation of violet-positive cells was analyzed by flow cytometry and suppressive functionality index was determined by calculating dividing cells (div)/ non-dividing (ndiv) cells (F). * p<0.05, Mann Whitney test. ASA = allogeneic stimulation assay, scGOS/lcFOS = short chain galacto-, long chain fructo-oligosaccharides, MoDC = monocyte-derived dendritic cells, div = dividing cells, ndiv = non-dividing cells.

These induced CD4^+^Foxp3^+^ T cells differentiated by scGOS/lcFOS-stimulated MoDC showed in suppression assays an increased capacity to inhibit the proliferation of CD4^+^ responder cells (Fig 7A and 7E) compared to T cells induced by medium-/LPS-treated MoDC and Treg control (Fig 7B–7D). As demonstrated in Fig 7F these scGOS/lcFOS-induced Foxp3^+^ T cells are characterized by a low suppressive functionality index comparable to Treg control which indicates a high capacity to inhibit the proliferation of CD4^+^ responder cells.

## Discussion

It has been reported in animal trials and human clinical studies that NDO like scGOS/lcFOS supplemented to infant formula can contribute to a decreased susceptibility to atopy and allergy [16,28–30]. However, the exact mechanisms are still unclear but might involve the direct effects of NDO on host-cells [31]. The specific mixture of scGOS/lcFOS, as used in this study, was previously found to modulate cytokine release from CD3/CD28 stimulated PBMC both directly and through effects on epithelial cells in 2D co-cultures [32], which suggest that scGOS/lcFOS might stimulate (immune) cells directly. In this study, we evaluated whether scGOS/lcFOS exert direct immune-modulatory effects on human MoDC. For the first time we show direct, immune-modulatory effects of this unique combination of oligosaccharides on human MoDC and the ensuing induced T cell response. We have shown that DC treated with scGOS/lcFOS released IL-10 in the absence of IL-12p70 and that this effect is mediated via activation of TLR4. In contrast, TLR4 activation through LPS stimulation of MoDC released both IL-10 and IL-12, which would argue against a potential bacterial product contamination of the scGOS/lcFOS preparation. In line with this, upon measuring the endotoxin content of the scGOS/lcFOS preparation using a LAL-assay, endotoxin content was measured to be very low (<3 ng/ml). Moreover, upon analysis of additional soluble mediators (chemokines as well as cytokines) the profile of scGOS/lcFOS induced release was markedly different from LPS, suggesting that other PRR may play a role.

Several studies describe an immune modulation by different NDO on cellular level via the interaction with specific sugar receptors including DC-SIGN, TLRs or C type lectin receptors [24,25,33–35]. Further studies in our experimental setting are necessary to investigate the role of other types of receptors involved in scGOS/lcFOS-mediated, direct mechanisms on human MoDC. Synbiotics, which are a specific mixture of probiotic bacteria and prebiotic NDO, can be ingredients of infant milk formulas [36]. Here the prebiotics are thought to act as substrates for the probiotic bacteria thereby assisting in their probiotic function. Strains from *Bifidobacteria* and *Lactobacilli* are often considered as potential probiotic bacteria [37]. Here we evaluated the contribution of the prebiotic mixture scGOS/lcFOS on the probiotic bacteria-induced MoDC cytokine profile. MoDC strain-dependently released IL-10 and IL-12. This is in accordance with previous data showing that different bacterial strains have different, strain-dependent effects on cytokine release of human immune cells potentially through the differential use of PRR [38–41]. In line with the data observed on the direct effects of scGOS/lcFOS on MoDC, the scGOS/lcFOS mixtures dose-dependently enhanced *B*.*breve* and *L*.*rhamnosus* induced IL-10 release while IL-12 release was unaffected. Again, this effect could be blocked using a LPS antagonist indicating a role for TLR4 in the observed effect. DC production of IL-10 in the absence of IL-12p70 could contribute to the development of regulatory T cells [27,42–44]. Additionally, treatment with oligosaccharides only slightly upregulated maturation markers CD80, CD83 and CD86 in comparison to LPS-matured DC. It has been postulated that partially or semi-mature intestinal DC producing little IL-12 in the presence of IL-10 are involved in induction of tolerance [27,45]. Experiments of this study show that treatment of MoDC with scGOS/lcFOS led to an increase in Foxp3^+^ T cells, which is associated with an increase in Treg [46,47]. Treg induction by scGOS/lcFOS shown in this study is in accordance with previous studies where a diet containing scGOS/lcFOS increased vaccination responses in mice but also decreased allergic symptoms in a mouse model of food-allergy [48,49]. According to van der Aa et al. a combination of *B*.*breve* and scGOS/lcFOS induces Treg cell development through modulation of DC function, preventing asthma-like symptoms in infants with atopic dermatitis [50]. An intervention study in a mouse model for orally induced cow’s milk allergy revealed a reduced acute allergic skin response and anaphylactic scores after dietary intervention with scGOS/lcFOS in combination with *B*.*breve*. Also enhanced whey-specific Th1 type serum IgG2a, fecal IgA and reduced mast cell degranulation was found in this study [51]. The authors suggested a possible involvement of whey-specific CD25^+^ regulatory T cells in induction of tolerance by NDO and confirmed a role for regulatory T cells in suppressing casein allergy by NDO [49,52]. An important role of regulatory T cells as well as anti-inflammatory cytokine profiles in the prevention of allergies and related disorders has been affirmed by several studies [53–55]. Finally, the results of our study provide support that the clinical efficacy of scGOS/lcFOS displaying similar functionality as HMO supplemented to infant formula may also relate in part to direct interaction with immune cells. Our data provides evidence for a direct immune-modulating effect on human dendritic cells involving TLR4 and subsequently an induction of regulatory T cells that could lead to an attenuation or prevention of diseases associated with loss of tolerance.

## Supporting Information

S1 Fig Gating strategy by means of FMO control. MoDC stimulated with scGOS/lcFOS (5 mg/ml) were used to prime naïve CD4^+^CD45RA^+^ T cells in an ASA. At day 7, cells were stained for CD4 but not intracellular Foxp3 analyzed by flow cytometry. The gating strategy (with FMO control) is shown which was used to obtain percentages of CD4^+^Foxp3^+^ cells shown in Fig 6. CD4^+^Foxp3^+^ cells were derived out the gate of alive cells after excluding duplicates. scGOS/lcFOS = short chain galacto-, long chain fructo-oligosaccharides, ASA = allogeneic stimulation assay, FMO = fluorescence minus one, MoDC = monocyte-derived dendritic cells.
(TIF)

S2 Fig Oligosaccharides induce no full MoDC maturation compared to LPS. MoDC were stimulated with oligosaccharides (5 mg/ml), LPS or medium for 24h. MoDC matured by LPS served as positive control for functional stimulation. The mean fluorescence intensities (MFI) specific for maturation markers CD80 (A), CD83 (B), CD86 (C), CD40 (D) and HLA-DR (E) as well as cell viability (F; propidium iodide staining) were determined by flow cytometry. Three to five independent experiments are shown. * p<0.05, ** p<0.01, Mann Whitney test.
(TIF)

S3 Fig Effect of *B*.*breve* on MoDC cytokine release is not affected in the presence of a TLR4 antagonist. Immature human MoDC were incubated with *B*.*breve* (1x10^6^cfu/ml) or LPS (100 ng/ml) in the presence or absence of a TLR4 antagonist (LPS-RS) for 24h. Medium- and TLR4 only-treated MoDC served as negative control, MoDC matured by LPS as positive control for functional MoDC stimulation. Results are presented as mean ± SEM, five independent experiments are shown. **** p<0.0001, ** p<0.01, unpaired student’s t test.
(TIF)

S4 Fig LAB *B*.*breve* and *L*.*rhamnosus* differ in their capacity to induce IL-12p70 secretion by human MoDC while IL-10 release is induced in a comparable and dose dependent manner. Immature human MoDC were stimulated with *B*.*breve* (dot) or *L*.*rhamnosus* (triangle) in different concentrations (1x10^5^-1x10^7^cfu/ml) for 24h. Amounts of IL-10 (A) and IL-12p70 (B) were measured by ELISA in cell-free supernatant. Results are presented as mean ± SEM, ten independent experiments are shown. * p<0.05, ** p<0.01, *** p<0.001, Mann Whitney test. LAB = Lactic acid bacteria, MoDC = monocyte-derived dendritic cells.
(TIFF)

S5 Fig scGOS/lcFOS enhance cytokine and chemokine release by LAB-stimulated MoDC. MoDC were stimulated with scGOS/lcFOS only or combinations of the mixture scGOS/lcFOS (5 mg/ml) and either *B*.*breve* or *L*.*rhamnosus* at a concentration of 1x10^5^ cfu/ml for 24h. The amount of cytokines and chemokines in cell-free supernatants was measured by luminex-based assay (readout: IL-1α, IL-1β, IL-6, IL-23p19, IL-27, TNFα, IP-10, MDC (CCL22), TARC (CCL17), MIP3α (CCL20) and ELISA (IL-10, IL-12p70)). Results are presented as induction factors that were obtained by dividing cytokine release of scGOS/lcFOS+LAB-stimulated MoDC through cytokine secretion of MoDC incubated with scGOS/lcFOS only.
(TIFF)

S6 Fig Gating strategy by means of CD4^+^ responder only control. T cells of allogeneic stimulation assays (ASA) were harvested on day seven and co-incubated with freshly isolated, violet-labeled responder CD4^+^ T cells a ratio of 1:1 for five days in presence of anti-CD3/28 and rhIL-2. The mix of ASA cells and violet stained responder CD4^+^ T cells was then stained (after five days of co-incubation) with CD4 APC-Cy7. The gating strategy (obtained by CD4^+^ responder cells only) is demonstrated which was used to calculate the suppressive functionality index shown in Fig 7. Dividing T cells (P4) and non-dividing T cells (P3) were derived out of the CD4^+^ gate. ASA = allogeneic stimulation assay.
(TIF)
