# Supplementary figures and images for: In Vitro Evidence for Immune-Modulatory Properties of Non-Digestible Oligosaccharides: Direct Effect on Human Monocyte Derived Dendritic Cells
Source: PLoS One. 2015 Jul 6;10(7):e0132304. doi: 10.1371/journal.pone.0132304 (PMC4493044; doi:10.1371/journal.pone.0132304)

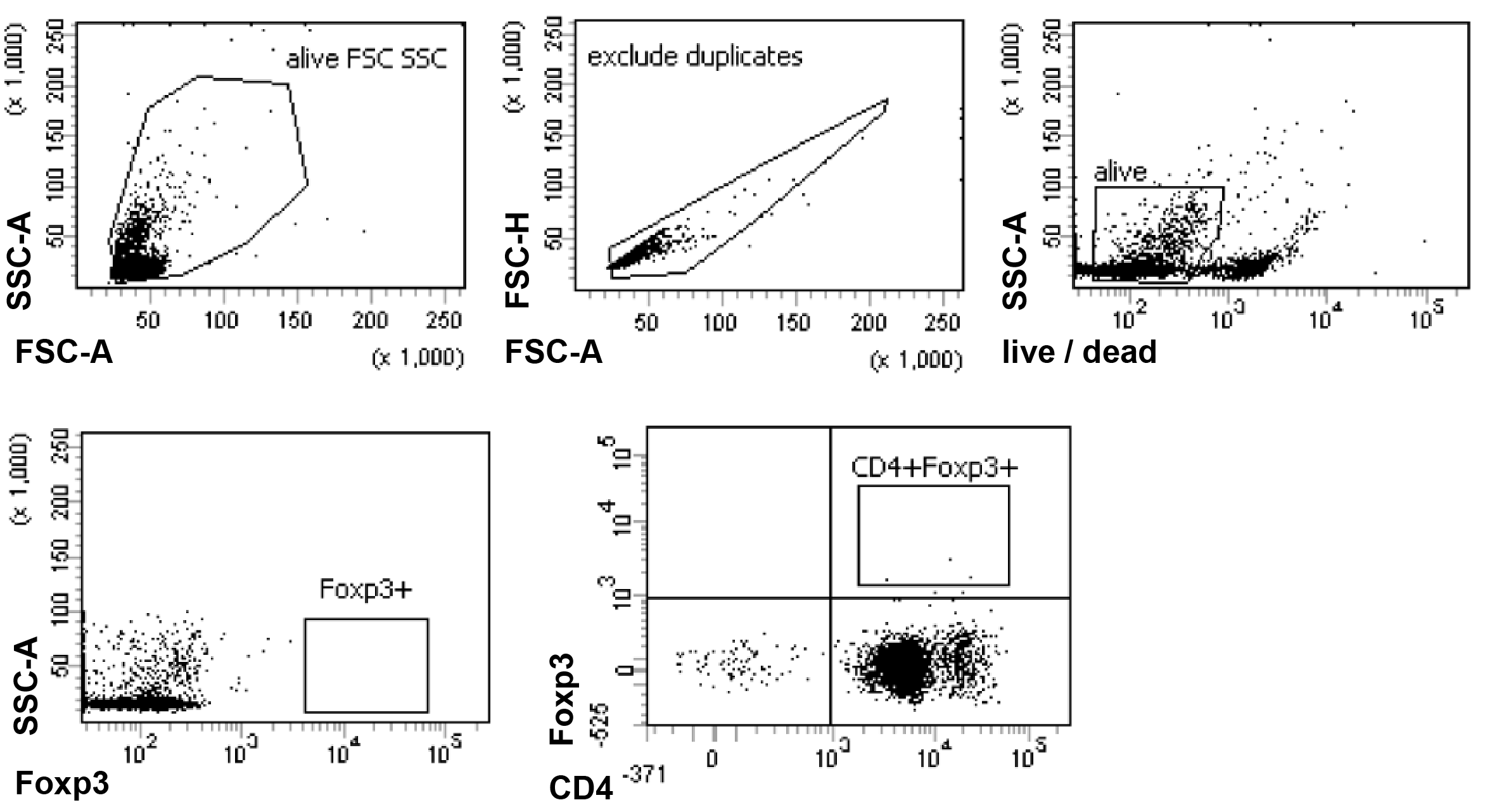

Supplement: S1 Fig — MoDC stimulated with scGOS/lcFOS (5 mg/ml) were used to prime naïve CD4+CD45RA+ T cells in an ASA. At day 7, cells were stained for CD4 but not intracellular Foxp3 analyzed by flow cytometry. The gating strategy (with FMO control) is shown which was used to obtain percentages of CD4+Foxp3+ cells shown in Fig 6. CD4+Foxp3+ cells were derived out the gate of alive cells after excluding duplicates. scGOS/lcFOS = short chain galacto-, long chain fructo-oligosaccharides, ASA = allogeneic stimulation assay, FMO = fluorescence minus one, MoDC = monocyte-derived dendritic cells. (TIF) [file pone.0132304.s001.tif]

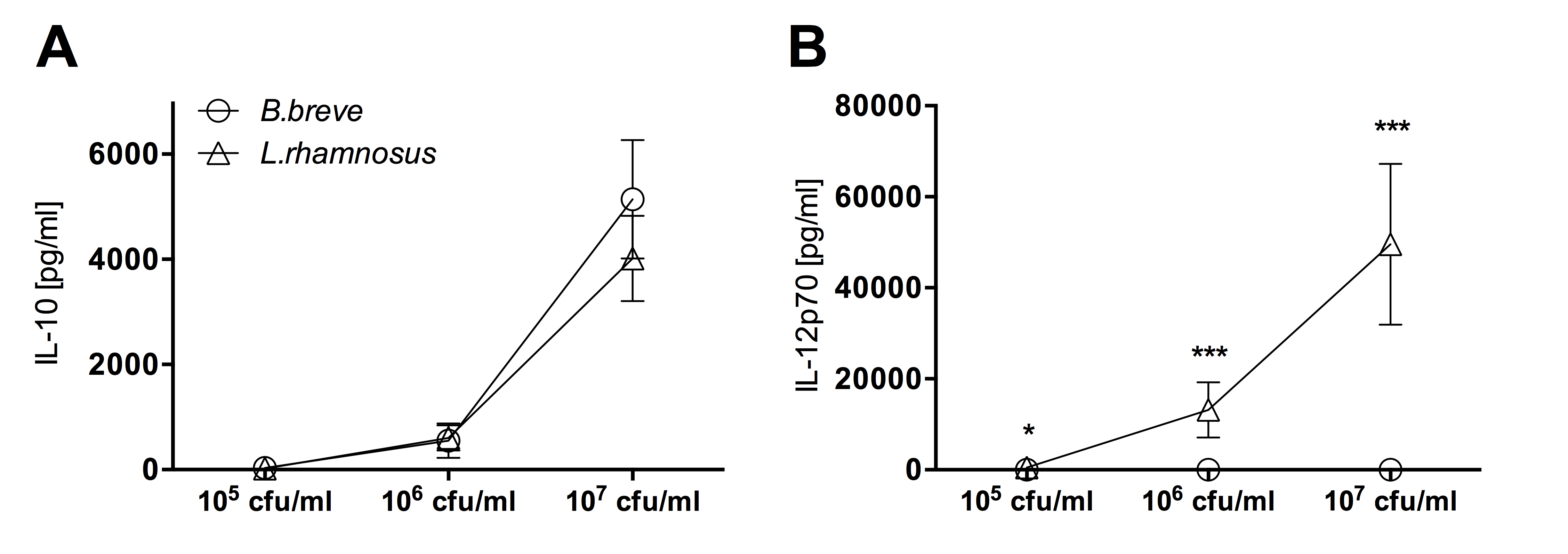

Supplement: S4 Fig — Immature human MoDC were stimulated with B.breve (dot) or L.rhamnosus (triangle) in different concentrations (1x10^5^-1x10^7^cfu/ml) for 24h. Amounts of IL-10 (A) and IL-12p70 (B) were measured by ELISA in cell-free supernatant. Results are presented as mean ± SEM, ten independent experiments are shown. * p<0.05, ** p<0.01, *** p<0.001, Mann Whitney test. LAB = Lactic acid bacteria, MoDC = monocyte-derived dendritic cells. (TIFF) [file pone.0132304.s004.tiff]

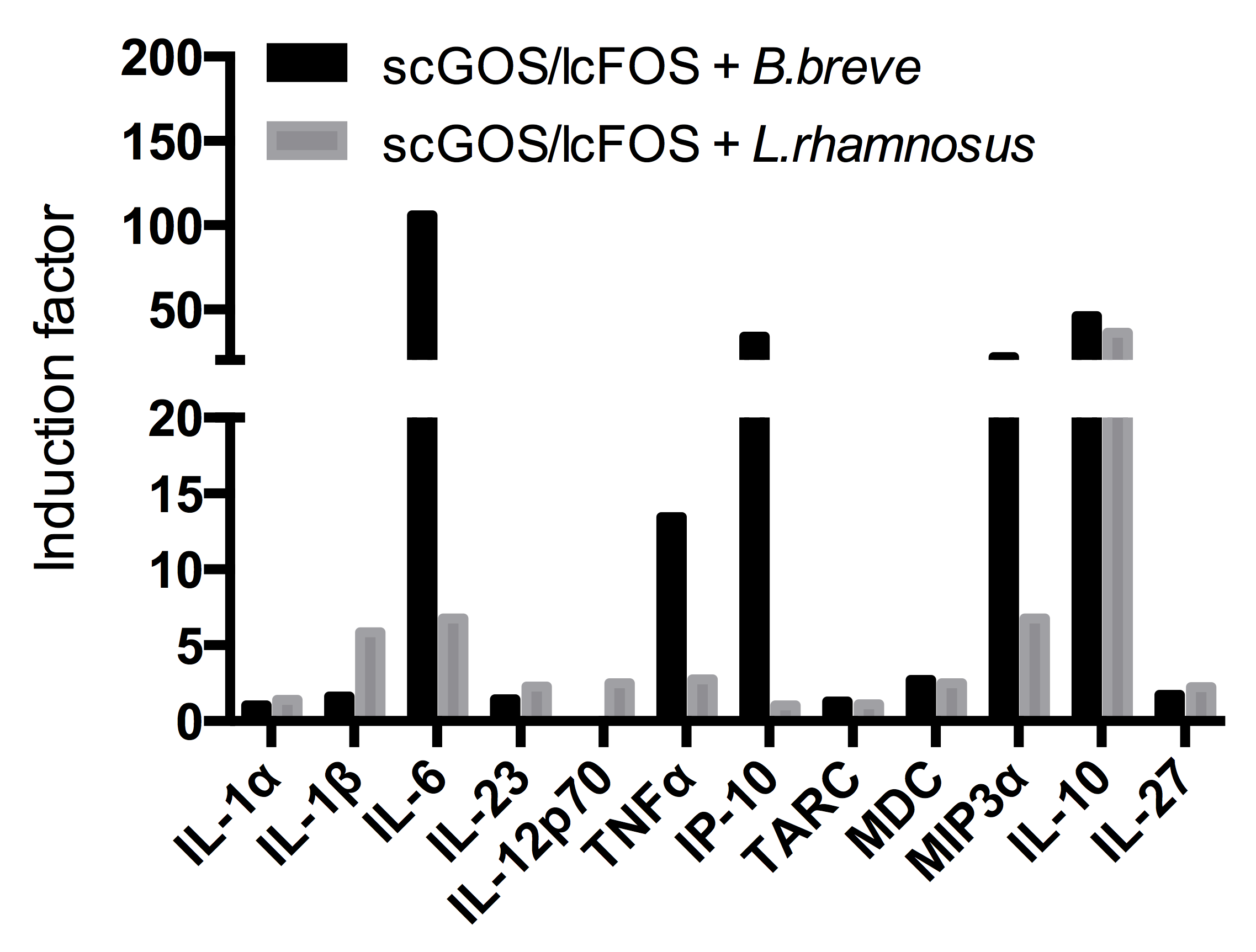

Supplement: S5 Fig — MoDC were stimulated with scGOS/lcFOS only or combinations of the mixture scGOS/lcFOS (5 mg/ml) and either B.breve or L.rhamnosus at a concentration of 1x10^5^ cfu/ml for 24h. The amount of cytokines and chemokines in cell-free supernatants was measured by luminex-based assay (readout: IL-1α, IL-1β, IL-6, IL-23p19, IL-27, TNFα, IP-10, MDC (CCL22), TARC (CCL17), MIP3α (CCL20) and ELISA (IL-10, IL-12p70)). Results are presented as induction factors that were obtained by dividing cytokine release of scGOS/lcFOS+LAB-stimulated MoDC through cytokine secretion of MoDC incubated with scGOS/lcFOS only. (TIFF) [file pone.0132304.s005.tiff]

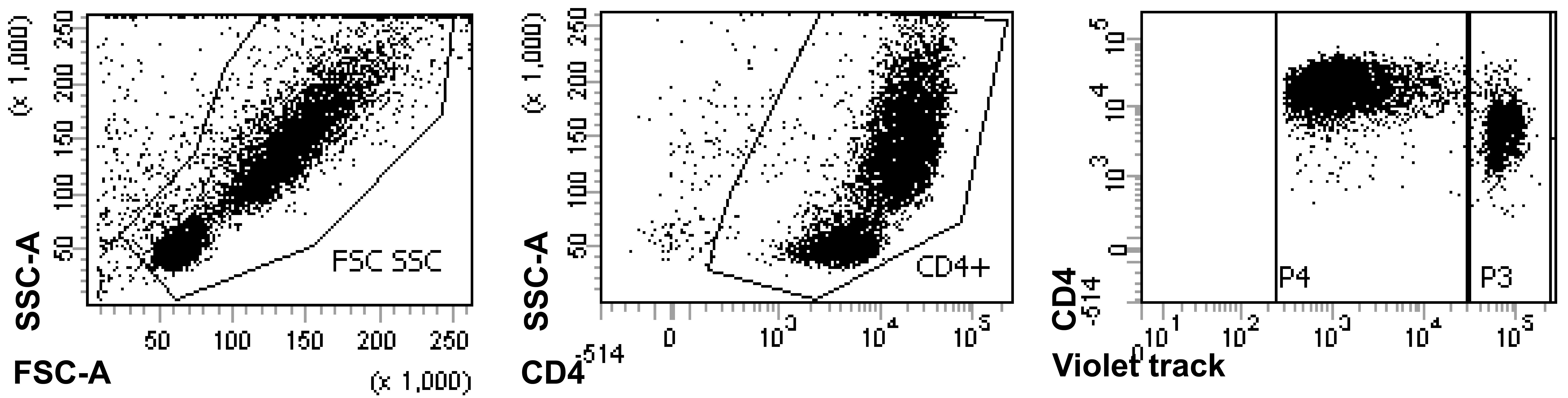

Supplement: S6 Fig — T cells of allogeneic stimulation assays (ASA) were harvested on day seven and co-incubated with freshly isolated, violet-labeled responder CD4+ T cells a ratio of 1:1 for five days in presence of anti-CD3/28 and rhIL-2. The mix of ASA cells and violet stained responder CD4+ T cells was then stained (after five days of co-incubation) with CD4 APC-Cy7. The gating strategy (obtained by CD4+ responder cells only) is demonstrated which was used to calculate the suppressive functionality index shown in Fig 7. Dividing T cells (P4) and non-dividing T cells (P3) were derived out of the CD4+ gate. ASA = allogeneic stimulation assay. (TIF) [file pone.0132304.s006.tif]
